# Genetic and molecular approaches for patients with familial hemophagocytic lymphohistiocytosis: a multi-center experience from Mexico

**DOI:** 10.3389/fimmu.2026.1849014

**Published:** 2026-07-03

**Authors:** Arturo Gutiérrez-Guerrero, Saul O. Lugo-Reyes, Daniela Olivia López-Rivera, Jacqueline Sánchez-Herrera, Lucero Valenzuela-Vázquez, Mercy Estevez-Mieres, Maria Enriqueta Nuñez Nuñez, Beatriz Bayardo-Gutiérrez, Juan Carlos Lona-Reyes, Rosa Margarita Cruz-Osorio, Veronica Soto-Chavez, Martín Bedolla-Barajas, José Alonso Gutierrez-Hernández, Tania Barragan-Arevalo, Maria Fernanda Hidalgo-Martinez, Liliana Gomez-Cardenas, Diego Sierra-Muñoz, Samantha Perea Alvarez, Edna Venegas-Montoya, Aide Tamara Staines-Boone, Maria del Carmen Zarate-Hernández, Vania Maria Miranda-Saavedra, Gabriel Emmanuel Arce-Estrada, Selma Scheffler-Mendoza, Juan Carlos Bustamante-Ogando, Beatriz Adriana Llamas-Guillén, Miguel Ruiz-Fernández, Perla Veronica Reynoso-Arenas, Martín Eduardo Flores-Munguía, Carlos Torres-Lozano, Miguel Angel Bonal-Pérez, Estefany Graciela Mamani-Velásquez, Rubén Martínez-Barricarte, Rosa María Nideshda Ramírez-Uribe, Nina Pastor, Ivan Martinez-Duncker, Paul Gaytan, Jorge A. Yañez, Marco Antonio Yamazaki-Nakashimada, Christelle Lenoir, Sylvain Latour, Sara Elva Espinosa, Mario Ernesto Cruz-Munoz

**Affiliations:** 1 Laboratorio de Inmunodeficiencias, Secretaría de Salud, Instituto Nacional de Pediatría, Ciudad de México, Mexico; 2 Facultad de Medicina, Universidad Autónoma del Estado de Morelos, Cuernavaca, Morelos, Mexico; 3 Division de Pediatría, Departamento de Alergia e Inmunología Clínica Pediátrica, Nuevo Hospital Civil de Guadalajara “Doctor Juan I. Menchaca”, Guadalajara Jalisco, Mexico; 4 Departamento de Alergía e Inmunología, Hospital Infantil de México Federico Gómez, Ciudad de México, Mexico; 5 Departamento de Genética Medica, Hospital Infantil de México Federico Gómez, Ciudad de México, Mexico; 6 Servicio de Alergia e Inmunología Instituto Mexicano del Seguro Social, Hospital Regional General Número 1 (HGR No. 1), Culiacán, Sinaloa, Mexico; 7 Unidad Médica de Alta Especialidad Instituto Mexicano del Seguro Social Número 25 (IMSS No. 25), Monterrey, Nuevo León, Mexico; 8 Hospital Universitario, Universidad Autónoma de Nuevo León, Monterrey, Nuevo León, Mexico; 9 Departamento de Inmunología, Instituto Nacional de Pediatría, Ciudad de México, Mexico; 10 Hospital del Niño Morelense, Cuernavaca, Morelos, Mexico; 11 Hospital General Centro Médico Nacional (CMN) La Raza IMSS, Ciudad de México, Mexico; 12 Departamento de Pediatría, Centenario Hospital Miguel Hidalgo, Aguascalientes, Aguascalientes, Mexico; 13 Departamento de Inmunología Clínica y Alergía, UMAE-CMNO-IMSS, Guadalajara, Jalisco, Mexico; 14 Departamento de Pediatría, Undidad Médica de Alta Especialidad-Centro Médico Nacional de Oriente-Instituto Mexicano del Seguro Social (UMAE-CMNO-IMSS), Guadalajara, Jalisco, Mexico; 15 Departamento de Pediatría, Hospital Materno Infantil-Caja Nacional de Salud (CNS), La Paz, Bolivia; 16 Division of Genetic Medicine, Department of Medicine, Vanderbilt Genetics Institute, Vanderbilt University Medical Center, Nashville, TN, United States; 17 Department of Pathology, Microbiology, and Immunology, Vanderbilt Center for Immunobiology, Vanderbilt Institute for Infection, Immunology, and Inflammation, Vanderbilt University Medical Center, Nashville, TN, United States; 18 Unidad de Trasplante de Progenitores Hematopoyéticos y Terapia Celular, Instituto Nacional de Pediatría, Ciudad de México, Mexico; 19 Centro de Investigación en Dinámica Celular, Instituto de Investigaciones en Ciencias Básica y Aplicadas (IICBA), Universidad Autónoma del Estado de Morelos, Cuernavaca, Morelos, Mexico; 20 Instituto de Biotecnología, Universidad Nacional Autónoma de México, Cuernavaca, Morelos, Mexico; 21 Laboratory of Lymphocyte Activation and Susceptibility to Epstein Barr Virus (EBV), Inserm Unité Mixte de Recherche (UMR) U1163 Institute IMAGINE, Université Paris Cité, Paris, France

**Keywords:** hemophagocytic lymphohistiocytosis (HLH), inborn errors of immunity, innate immunity, next generation (deep) sequencing (NGS), NK cells

## Abstract

**Introduction:**

Hemophagocytic lymphohistiocytosis (HLH) is a life-threatening condition that results from a severe inflammation caused by an exaggerated immune response. HLH may have a genetic cause affecting the proper function of cytotoxic immune cells or it canbe linked to other pathological settings including inborn errors of immunity, malignancies, autoinflammatory and autoimmune syndromes, metabolic diseases, or acquired immunodeficiencies. HLH due to a genetic error remains difficult to diagnose because anormal Natural Killer (NK) or cytotoxic T lymphocyte (CTL) function does not necessarily exclude a familial form of HLH affecting immune cells other than cytotoxic lymphocytes, or because patients with autoimmune or autoinflammatory syndromes can also fulfill the HLH criteria. In consequence, sensitive functional assays and assessment of the expression of proteins involved in lytic-granules exocytosis may be useful approaches for discriminating between familial forms of HLH from those where a genetic cause is not affecting cytotoxic cell function, or from acquired forms of HLH.

**Methods:**

By different approaches, including functional assays and biochemical studies, we were able to obtain molecular diagnostics for patients, while next-generation sequencing identified the disease-associated gene variants.

**Results:**

Here we presented a multi-center experience in approaching and diagnosing patients with familial HLH. Our study included a cohort of 31 patients that fulfill the criteria of HLH. Genetic testing, led to identified variants in *PRF1, UNC13D, STX11, STXBP2, RAB27A, LYST, AP3B1* and *SH2D1A*, confirming most of the initial molecular diagnoses. From allfound variants, 15 were novel and not previously reported in patients with HLH nor found in homozygous condition.

**Discussion:**

Our results highlight the importance of taking into consideration molecular studies to increase the proportion of patients that obtain a molecular diagnosis, especially in countries where high-throughput genetic analyses are difficult to access or time-consuming.

## Introduction

Hemophagocytic lymphohistiocytosis (HLH) is a life-threatening condition that results from a severe inflammation caused by an exaggerated immune response. This excessive immune response is characterized by lymphoproliferation of strongly activated CD8+ T cells that produce high levels of IFN-γ leading to the secondary activation of macrophages (1). A typical clinical presentation of HLH includes prolonged fever, hepatosplenomegaly, and pancytopenia. In addition, laboratory findings comprise increased ferritin, triglycerides, bilirubin, soluble interleukin-2 receptor α-chain, and decreased fibrinogen (2–4). HLH may have a genetic cause affecting one or more immune cell type functions or can also be associated with inborn errors of immunity and other pathological settings including malignancies, autoinflammatory and autoimmune syndromes, metabolic diseases, or acquired immunodeficiencies (5). Furthermore, HLH has also been described in patients under iatrogenic scenarios including organ or stem cell transplantation or several immunotherapies.

Genetic studies have been pivotal in identifying monoallelic variants as causative for HLH. Initial studies discovered a group of genes implicated in regulating the exocytosis and function of perforin-containing lytic granules (6–10). Therefore, when a genetic defect affecting the proper function of NK cells and cytotoxic CD8+ T cells is identified as the leading cause, HLH is also referred to as familial HLH (FHL). In consequence, a defective NK cell function is also a characteristic finding in those patients suffering from FHL (11). Importantly, herpesvirus infections often by EBV or CMV are recognized as a prior condition to trigger the onset of HLH clinical symptoms (1) although other pathogens including dengue may also trigger HLH (12, 13). There are five recognized forms of FHL (FHL1-5), and from these, four genes have been identified. The specific gene causing FHL1 has not yet been identified, although a potential gene locus in chromosome 19 has been described. Specific mutations in the gene encoding for perforin (*PRF1*) are responsible for FHL2 (6), whereas mutations in *UNC13D* (7), *STX11* (8), and *STXBP2* (10) account for FHL3 through 5 respectively. HLH is also observed in syndromes with defective pigmentation and increased susceptibility to infections. In these entities, variants in *RAB27A* (14, 15), *LYST* (16, 17) and *AP3B1* (18) were identified as the genetic leading causes for Griscelli syndrome type 2 (GS2) II, Chediak-Higashi syndrome (CHS), and Hermansky-Pudlak Syndrome (HPS) respectively. All these genes encode for proteins involved in the trafficking and exocytosis of perforin-containing granules in cytotoxic lymphocytes. A third syndrome characterized by a life-threatening HLH selectively triggered by EBV infection led to the identification of variants in *SH2D1A* (19–21), a gene that encodes for the SAP protein, an adapter molecule that allows signaling by the SLAM family receptors. All these discoveries have been fundamental for establishing a close relation between an impaired perforin-mediated cytotoxicity and the development of HLH, notably in the context of EBV infection in patients with *SH2D1A* mutations. Moreover, they were fundamental for achieving a deep understanding of the cellular and molecular mechanisms that predispose to HLH. Besides the initial descriptions of genetic variants affecting perforin-mediated cytotoxicity, other genetic entities were shown to contribute to HLH susceptibility. High-throughput sequencing allowed the identification of variants in *RHOG* (22), *CDC42* (23), and *NRLC4* (24), among others (25). However, in contrast to genetic variants affecting NK and CD8+ T cells function, all these recently described genes apart from *RHOG* play a role in immune cells other than cytotoxic lymphocytes. Moreover, patients manifest HLH with different degrees of penetrance depending on the affected gene. All these studies suggest that the pathophysiology of HLH has a more complex picture involving molecules other than perforin and IFN-γ.

In 1991 the diagnostic guidelines for HLH were published by the HLH Study Group of the Histiocyte Society. The guidelines included eight clinical and laboratory criteria including a decreased or absent NK cell function. Five of eight of these criteria are sufficient to fulfill the diagnosis of HLH (4). Despite all advances in the diagnosis of the more frequent FHL, HLH still remains difficult to diagnose because a normal NK or CTL function does not necessarily exclude a genetic form of HLH, or because patients with of autoimmune or autoinflammatory syndromes can also fulfill the HLH criteria. Since a prompt HLH diagnosis determines the patient treatment and outcome, it becomes necessarily important to use all accessible tools that may help to discern between familial forms of HLH due to a failure of cytotoxic lymphocytes from HLH due to other genetic errors or from HLH observed in autoinflammatory or autoimmune settings. This becomes relevant, especially in those countries where next generation sequencing is not always a readily available tool. Therefore, sensitive functional assays and determination of protein expression may be useful approaches to complement high-throughput genetic analyses. Such efforts can hopefully substantially increase the proportion of patients that obtain a molecular diagnosis. Here we presented a cohort of 31 patients that presented HLH as the major clinical manifestation. By different approaches, including functional assays and biochemical studies, we were able to obtain molecular diagnoses for patients. Genetic testing, by next generation sequencing, confirmed all initial molecular diagnoses.

## Materials and methods

### Cell culture

Peripheral blood samples were collected after obtaining informed consent under a local Institutional Review Board-approved study investigating the genetic causes of hemophagocytic lymphohistiocytosis (HLH) syndrome. Human peripheral blood mononuclear cells (PBMCs) derived from patients or age non-matched healthy controls were isolated by density gradient centrifugation using Ficoll-Hypaque (GE Healthcare Life Sciences). PBMCs, as well as the K562 and the P815 cell lines (both obtained from ATCC) were cultured in Advanced RPMI-1640 medium (Invitrogen Life Technologies) supplemented with 5% (v/v) FBS (Byproducts), 100 U/mL penicillin, 0.1 mg/mL streptomycin, L-glutamine, and 2-mercaptoethanol (Invitrogen Life Technologies). Cells were incubated at 37 °C in humidified atmosphere containing 5% CO_2_.

### Degranulation assays

To assess NK-cell degranulation, PBMCs were cultured with an equal number of K562 cells (2 × 10^5^) in 96-well U-bottom plates in a total volume of 200 µl. Plates were centrifuged at 500 rpm for 3 minutes to allow conjugate formation and incubated at 37 °C in humidified atmosphere containing 5% CO_2_ for 3 hours. In some samples, PBMCs were co-cultured with P815 cells coated with an isotype control, anti-CD16 antibody (3G8), or anti-CD3 antibody (OKT3) at 1:1 effector-to-target (E:T) cell ratio. For the assessment of CD8^+^ T-cell degranulation, PBMCs were pre-activated with PHA (Sigma-Aldrich) for 48 hours prior to E:T cell co-culture to induce cytotoxic T lymphocytes. The cells were then harvested and surface-stained with antibodies to FITC-anti-CD3 (OKT3), APC-anti-CD56 (5.1H11) or APC-anti-CD8a (HIT8a), and PE-anti-CD107a (H4A3) at 4 °C in the dark for 30 minutes. All antibodies were purchased from BioLegend. Degranulation of NK-cells (defined as CD3^−^CD56^+^) or CD8^+^ T-cells (defined as CD3^+^CD8a^+^) was determined by quantifying the frequencies of CD107a-expressing cells at the cell surface after stimulation with K562 cells, P815 cells, or medium alone. Samples were acquired on a three-laser BD FACSCanto II flow cytometer, and data were analyzed using FlowJo software (version 10, TreeStar).

### SAP intracellular expression

For the evaluation of intracellular expression of SAP in T lymphocytes, the PBMCs were stained with the following panel of fluorochrome-conjugated monoclonal Abs directed against cell surface markers: CD3 FITC (OKT3), CD4 PerCP Cy5.5 (OKT4), CD19 APC (HIB19). Then, cells were fixed and permeabilized using Cytofix/Cytoperm Kit (BD Bioscience) and finally stained using the murine PE conjugated monoclonal antibody directed against human SAP (XLP 1D12). The PE signal in B cells was used as a staining control for SAP expression. Flow cytometric data were acquired on FACSCanto II (BD bioscience) and analyses with the use of FlowJo 7.6.5 software (TreeStar).

### Western blotting

Equal numbers of expanded PBMCs were lysed in TNE buffer (50 mM Tris [pH=8.0], 1% NP-40, 2 mM EDTA) supplemented with a proteinase inhibitor cocktail (Roche) for 30 minutes on wet ice. Cell debris was removed by centrifugation and the supernatants were recovered. The cell lysates were separated by reducing SDS-PAGE and subsequently transferred onto polyvinylidene difluoride membrane (Millipore). The membranes were blocked with 5% non-fat dry milk for 1 hour in LCK buffer (1 M tris-base pH 7.5, 0.5M EDTA pH 8.5, 0.05% Tween 20, and 50 mM NaCl), then incubated with primary antibodies (anti-Rab27a or anti-β-actin; Cell Signaling Technology) followed by HRP-conjugated secondary antibodies (Santa Cruz Biotechnology). Blots were developed with ECL and exposed to film (Kodak).

### RT-PCR and sequencing

Total RNA from PBMCs of both the patients and healthy controls was extracted using TRIzol™ reagent (Invitrogen Life Technologies) according to the manufacturer´s instructions. Reverse transcription was performed using an ImProm-II™ Reverse Transcription System (Promega) and cDNAs were used as templates to amplify the *RAB27A* gene by PCR with specific primers for encoding exons. The amplified PCR products were sequenced bidirectionally using dye-terminator chemistry.

### Whole-exome sequencing

Genomic DNA was extracted from whole peripheric blood or isolated PBMCs and analyzed via Whole-exome sequencing as described elsewhere (26).

### Statistical analysis

Statistical analysis was performed using an unpaired Student´s *t* test (two-tailed). All statistical calculations were performed using Prisma software (version 9.0.0, GraphPad).

## Results

**Figure 1:**
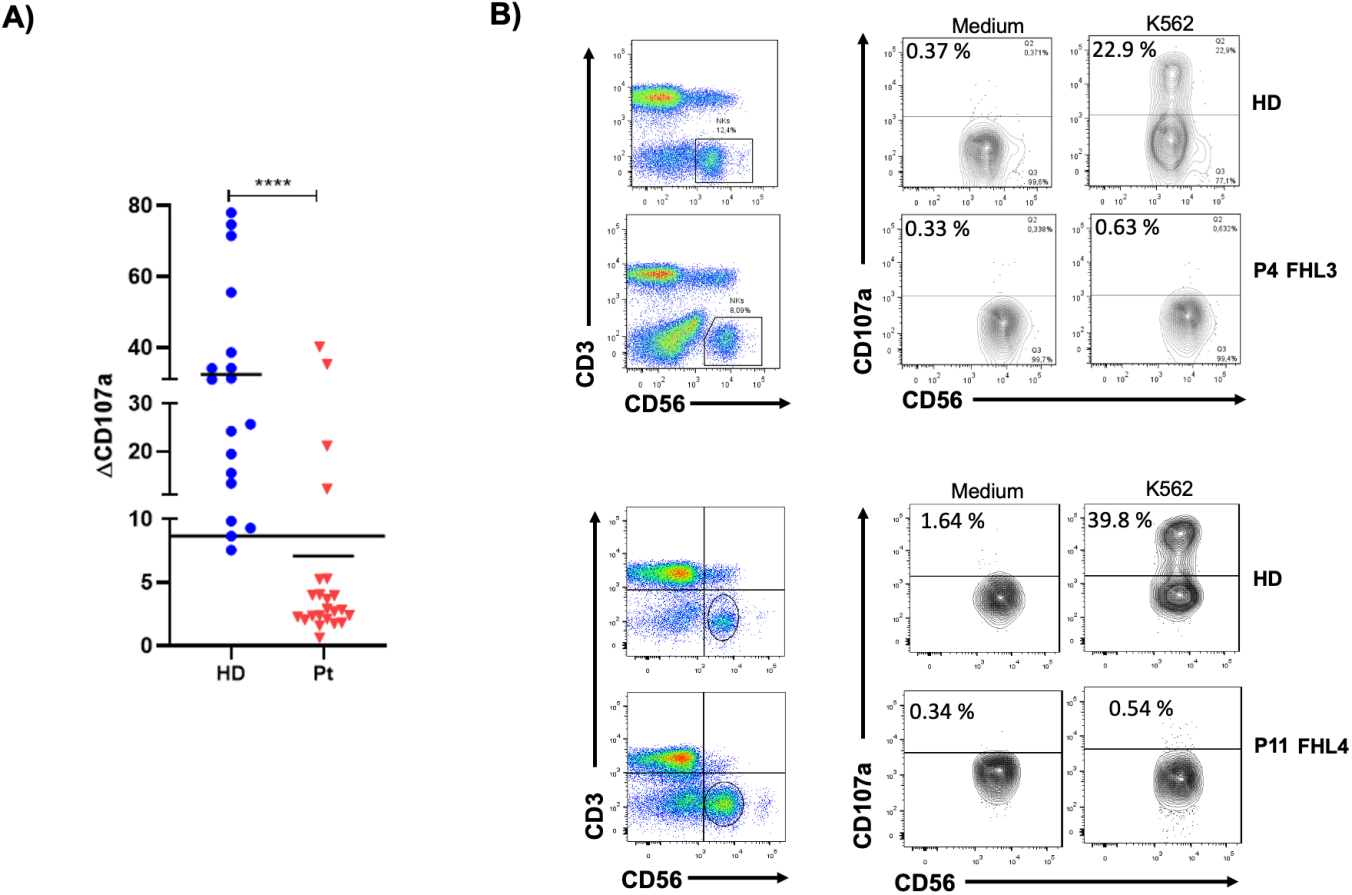
Evaluation of NK cell degranulation in patients with clinical manifestations of familial hemophagocytic lymphohistiocytosis (FHL). **(A)** Statistical analysis of NK cell degranulation assays from eighteen healthy donors (HD; blue) and twenty-three patients (Pt; red). Horizontal lines represent mean values, and *p* values <0.05 were considered statistically significant. ΔCD107a indicates the difference in the percentage of cells expressing CD107a before stimulation subtracted from the percentage of cells expressing CD107a after stimulation. The threshold for degranulation was defined based on the 10^th^ percentile of HD. **(B)** FACS plots illustrating the expression of CD107a on NK cell surface (CD3-CD56+) using PBMCs from a healthy donor (HD) or a patient with FHL3 (P4, upper panel) or a patient with FHL4 (P11, bottom panel) after incubation with medium or with NK-sensitive K562 target cells. Data are presented as the percentage of NK cells expressing surface CD107a.

During a period of four years, we received samples from thirty-one patients that fulfill five of the eight criteria of HLH according to the HLH-2004 guidelines (2). All patient samples were obtained from different tertiary hospitals belonging to different regions of Mexico. To obtain differential diagnosis of familial forms of HLH due to an impaired NK cell and CTL function, we performed degranulation assays based on the quantification of CD107a on the surface of cytotoxic lymphocytes, as has been previously reported (11). Degranulation assays were possible in twenty-four patients. Except where indicated, all assays were performed using resting NK cells (not treated with IL-2). The NK cell-sensitive K562 cell line was used as target cells unless otherwise indicated. A defective NK cell degranulation was defined when the value for ΔCD107a was below 8.5 according to the 10^th^ percentile of healthy donors (see statistical analysis). NK degranulation assays led to identification of nineteen patients with a defective NK cell function whereas four patients (P14, P29, P30, P31) displayed normal NK cell activity (**Figure 1A**). For only one patient (P27, not included in **Figure 1A**), it was not possible to use K562 cells, instead a reverse Antibody Dependent Cell Cytotoxicity (ADCC) assay was used to assess NK cell function. During these functional assays, no genetic testing was performed for most of the patients. Further whole exome sequencing (WES) revealed that of nineteen patients with a defective NK cell degranulation, thirteen patients were carrying homozygous or compound heterozygous variants in *PRF1*, *UNC13D*, *STX11* and *STXBP2* (**Table 1**). **Figure 1B** shows a representative NK cell degranulation assay for patients with *UNC13D* (P4) and *STX11* (P11) deficiency. Two patients (P1 and P2) were found to carry variants in *PRF1*. P1 presented biallelic mutations (c.445G>A, p.Gly149Ser; c.755A>G, p.Asn252Ser) whereas P2 presented a homozygous mutation (c.445G>A, p.Gly149Ser). In addition, P1 was also a carrier for a heterozygous variant in *UNC13D*, which has been previously identified in heterozygous state in an individual with hemophagocytic lymphohistiocytosis (27). Both *PRF1* variants have also been reported in individuals with FHL2 (6, 28).

**Table 1:** Genetic variants, functional outcomes, and allele frequencies in 31 patients.

Patient	Gene	Variant	Experimental outcome	Allele frequency (gnomAD)
P1	*PRF1* *UNC13D*	c.755A>G (p.Asn252Ser) c.445G>A (p.Gly149Ser) c.71G>A (p.Arg24His)	Undetermined	0.005% 0.00014%/0.00064 Latino 0.03%
P2	*PRF1*	c.445G>A (p.Gly149Ser) Hom	Undetermined	0.00014%/0.00064 Latino
P3	*UNC13D*	c.859-3C>A Hom	Defective NK cell degranulation	0.02%
P4	*UNC13D*	c.1072dup (p.Ser358Lysfs*103) c.859del (p.Arg287Glufs*41)	Defective NK cell degranulation	No frequency No frequency
P5	*UNC13D*	c.570-2A>G c.2795T>C, (p.Leu932Pro)	Defective NK cell degranulation	0.01% 0.1%
P6	*UNC13D*	c.833T>C (p.Leu278Pro) Hom	Defective NK cell degranulation	No frequency
P7	*UNC13D*	c.1831G>C (p.Ala611Pro) Hom	Defective NK cell degranulation	No frequency
P8	*UNC13D*	c.1721C>G (p.Ser574Ter) Hom	Defective NK cell degranulation	0.003%
P9	*UNC13D*	c.859del (p.Arg287GlufsTer41) c.2795T>C (p.Leu932Pro)	Defective NK cell degranulation	No frequency 0.1%
P10	*STX11*	c.83C>A, p.S28* Hom	Undetermined	0.006%
P11	*STX11*	c.157_160del (p.Asp53LysfsTer9) Hom	Defective NK cell degranulation	0.001%
P12	*STXBP2*	c.284del (p.Pro95Argfs*24) c.560C>T (p.Pro187Leu)	Defective NK cell degranulation	0.003% 0.004%
P13	*STXBP2*	c.284del (p.Pro95Argfs*24) Het	Undetermined	0.003%
P14	*RAB27A*	c.343A>G (p.Ser115Gly)N Hom	Diminished NK cell degranulation	No frequency
P15	*RAB27A*	c.335del (p.Asn112Thrfs*3) Hom	Undetermined	0.03%
P16	*RAB27A*	c.335del (p.Asn112Thrfs*3) Het	Defective NK cell degranulation	0.03%
P17	*RAB27A*	c.335del (p.Asn112Thrfs*3) Hom	Undetermined	0.03%
P18	*RAB27A*	c.335del (p.Asn112Thrfs*3) Hom	Defective NK cell degranulation	0.03%
P19	*RAB27A*	Not found in encoding region	Defective NK cell degranulation	No frequency
P20	*RAB27A*	Not found in encoding region	Defective NK cell degranulation	No frequency
P21	*RAB27A*	Not found in encoding region	Defective NK cell degranulation	No frequency
P22	*RAB27A*	c.335del (p.Asn112Thrfs*3) c.333A>C (p.Arg111Ser)	Defective NK cell degranulation	0.03% No frequency
P23	*LYST*	c.1897A>T (p.Lys633*)/ c.6676C>T (p.Arg2226*)	Defective NK cell degranulation	0.01% 0.003%
P24	*LYST*	c.3574G>T (p.Glu1192*) Hom	Defective NK cell degranulation	No frequency
P25	*LYST*	c.10900G>A (p.Val3634Met) Het	Undetermined	No frequency
P26	*LYST*	c.9784+1G>T Hom	Defective NK cell degranulation	0.003%
P27	*LYST*	c.10222G>A (p.Gly3408Arg) Hom	Defective NK cell degranulation*	No frequency
P28	*AP3B1*	c.1679A>G (p.Asn560Ser)/ c.2018A>G (p.Lys673Arg)	Defective NK cell degranulation	0.08% 0.08%
P29	*SH2D1A*	c.293T>C (p.Leu98Pro) Hom	Normal NK cell degranulation	0.006%
P30	WCGS**	NA	Normal NK cell degranulation	NA
P31	WCGS**	NA	Normal NK cell degranulation	NA

*Indicates that functional assay was performed by reverse Antibody Dependent Cell Cytotoxicity (rADCC) and Patient was not included in **Figure 1A**.

**Indicates: Without Conclusive Genetic Study.

NA, Not applicable.

Nine *UNC13D* variants were identified in eight patients. P3 was a homozygous carrier for a splice variant c.859-3C>A. P4 was a carrier of two variants c.1072dup (p.Ser358LysfsTer103) and c.859del (p.Arg287GlufsTer41). The c.859-3C>A variant and the c.859del have been already identified in individuals with HLH (29, 30). The variant (p.Ser358LysfsTer103) resulted in a nonsense mutations and is expected to result in an absent or disrupted protein product. This variant is not present in population databases nor has been reported in individuals suffering from FHL3. NK cells from this patient showed defective NK cell degranulation (**Figure 1A**), suggesting that the variant (p.Ser358LysfsTer103) can be classified as likely pathogenic (**Figure 1B**). Variants c.570-2A>G and c.2795T>C (p.Leu932Pro) were identified in P5. Whereas the variant c.570-2A>G has been observed in individuals with FHL3 (30, 31), the p.Leu932Pro substitution has not been reported in *UNC13D*-related conditions, although it has a low frequency in population databases (rs760552006, gnomAD 0.1%). The change replaces leucine, which is neutral and non-polar, with proline, which is also neutral and non-polar, at codon 932 of the UNC13D protein (p.Leu932Pro). Modeling of protein sequence and biophysical properties indicates that this missense variant is expected to disrupt UNC13D protein function with a positive predictive value of 80%. Consistent with these *in silico* studies NK cells from the patient exhibited defective degranulation (**Figure 1A**), strongly supporting that this novel variant can be considered as likely pathogenic. Homozygous variants c.833T>C, (pLeu278Pro) and c.1831G>C (p.Ala611Pro) were identified in P6 and P7 respectively. Neither is found in any public database nor in individuals with UNC13D deficiency. Ala611 lies at the interface between two alpha helices in the MHD1 domain of UNC13D; placing a proline at this position would likely impair domain folding. This domain interacts with RAB27A and is crucial for recycling endosomes and lysosomes. On the other hand, Leu278 is packed in a hydrophobic core in one of the beta domains of UNC13D, adjacent to a twisted beta strand. Substitution by proline would not change the hydrophobic character of the site, but loss of a hydrogen bond donor at the backbone could destabilize the beta domain. A defective degranulation observed in NK cells from P6 and P7 suggests that the p.Ala611Pro and pLeu278Pro substitutions have a deleterious effect on NK cell function. P8 was carrier a homozygous variant c.1721C>G (p.Ser574Ter). This variant is present in population databases at a low frequency (gnomeAD 0.000016%; Hom=0), but has not been reported in patients with UNC13D. This mutation creates a premature translational stop signal (p.Ser574Ter) in the *UNC13D* gene and it is expected to result in an absent or disrupted protein product. NK cells from this patient displayed a defective NK cell degranulation (**Figure 1A**), suggesting that this not previously described variant is deleterious and causative. P9 was a heterozygous carrier for variants c.859del (p.Arg287GlufsTer41) and c.2795T>C (p.Leu932Pro), already identified in P4 and P5 respectively and consistent with a defective NK cell degranulation (**Figure 1A**).

Two distinct homozygous variants in *STX11* (c.83C>A, p.S28Ter and c.157_160del, p.Asp53LysfsTer9) were identified in two patients (P10 and P11 respectively) suffering from HLH. The variant c.83C>A (p.Ser28Ter) creates an early premature translational stop codon leading to short truncated protein that could result in the absence of protein expression. This variant is present at a weak frequency in population databases (rs143547259, gnomAD MAF = 0.00003% with no homozygous), but has not been reported in the literature in individuals with *STX11* deficiency. The mutation disrupts a region of the STX11 protein in which other variants (*e.g*., p.Leu58Pro) have been determined to be pathogenic (32, 33), which suggests that this is a clinically significant region of the protein, and variants that disrupt it are likely to be disease-causing. The variant c.157_160del (p.Asp53LysfsTer9) is observed at a very low frequency in the gnomAD dataset (MAF <0.001%) and is predicted to translate into a truncated protein shortened by more than 10%. This variant has not been previously reported in patients with *STX11* deficiency but has been classified as likely pathogenic. A defective NK cell degranulation assay of primary NK cells from patient with the p.Asp53LysfsTer9 mutant confirms its putative pathogenic role (**Figure 1B**, lower panel).

Patients 12 and 13 were carriers for variants in *STXBP2* gene. P12 presented two heterozygous variants, c.284del (p.Pro95ArgfsTer24) and c.560C>T (p.Pro187Leu). The frameshift variant c.284del (p.Pro95ArgfsTer24) creates a premature translational stop signal resulting in an absent or disrupted protein product. This variant is present in gnomAD (<0.003%) but has not been reported in patients with FHL5. The variant (p.Pro187Leu) has been already reported in individuals with clinical features of HLH (30). Impaired degranulation of NK cells from P12 (**Figure 1A**) thus strongly suggests that the variant p.Pro95ArgfsTer24 is also likely pathogenic and contributes along with the variant p.Pro187Leu for this defect. P13 presented the variant (p.Pro95ArgfsTer24) in heterozygous condition. The patient was diagnosed with systemic Juvenile Idiopathic Arthritis (sJIA). No other mutation in genes others than those causing FHL was identified for this patient.

**Figure 2:**
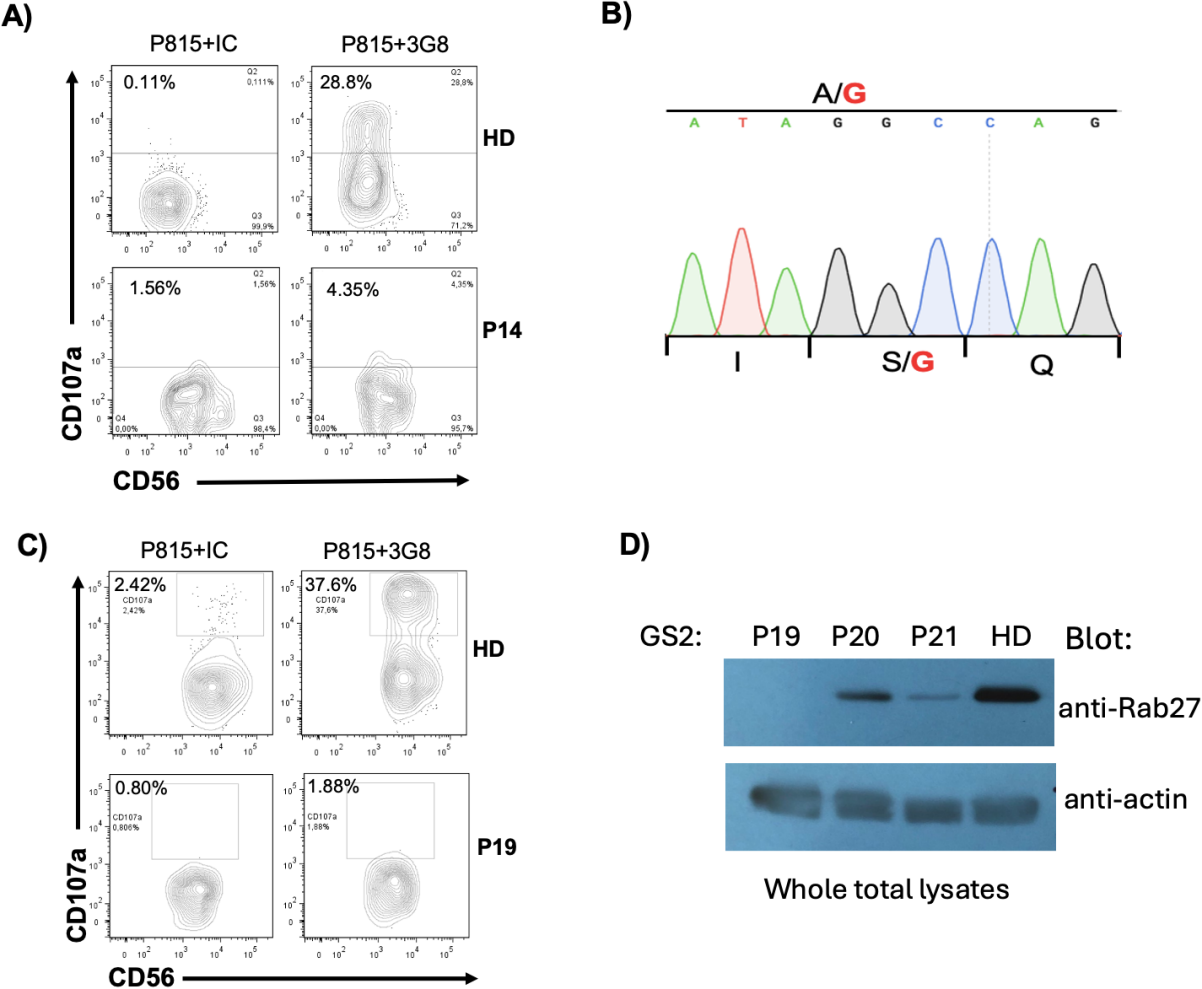
Evaluation of NK cell degranulation in patients with clinical manifestations of Griscelli Syndrome type 2 (GS2) and Chediak-Higashi syndrome (CHS). **(A)** FACS plots illustrating the expression of CD107a on NK cell surface (CD3-CD56+) using PBMCs from a healthy donor (HD) or from P14 after incubation with P815 cells in the presence of an isotype control (IC) or an anti-CD16 antibody (clone 3G8). **(B)** Sanger sequence revealed that P14 was a carrier for a homozygous missense variant in exon 4 of the *RAB27A* cDNA (c.343A>G, p.Ser115Gly). **(C)** FACS plots illustrating the expression of CD107a on NK cell surface (CD3-CD56+) using PBMCs from a healthy donor (HD) or from P19 after incubation with P815 cells in the presence of an isotype control (IC) or an anti-CD16 antibody (clone 3G8). **(D)** Immunoblot analysis of RAB27a protein expression and endogenous actin in PBMCs whole-cell lysates from P19, P20, P21 and a HD.

Nine patients (P14 to P22) had typical clinical characteristics of GS2 including (see **Supplementary Material** for major clinical manifestations). Seven of these nine samples were assessed for NK degranulation assays (**Figure 1A**). Six patients (P16, P18-P22) displayed a strong defect in NK cell degranulation that was almost completely abolished, whereas P14 presented a reduced NK cell degranulation. To have more clarity on NK cell function for this patient, we extended our studies by performing a reverse antibody dependent cell cytotoxicity assay (rADCC). This rADCC assay revealed that in contrast to NK cells from a healthy donor, NK cells of the patient displayed no difference in the frequencies for CD107-expressing NK cells between unstimulated and stimulated cells suggesting a degranulation defect (**Figure 2A**). At the time of NK cell function testing, no directed exome sequencing was possible for this patient. Therefore, by PCR, we amplified in four fragments, the *RAB27A*-specific fragment of the cDNA containing the last 5 exons (Exons 3-7). Sanger sequencing revealed that the *RAB27A* cDNA from this patient contained a homozygous mutation (**Figure 2B**). The mutation was an A to G transition at position 343 in the fourth exon. This point mutation resulted in a Ser to Gly substitution at residue 115. All these data together suggest that the p.Ser115Gly substitution can be deleterious for NK cell degranulation. The variant was not found in any public database nor reported in patients suffering from GS2.

A genetic study by directed-exome sequencing was possible for the other eight patients allowing the identification of variants in RAB27A in four patients (P15, P16, P17, P18 and P22). However, in three patients (P19-21), despite a defective NK cell degranulation being documented, no mutations were found in the encoding regions of *RAB27A* or other genes known to be causative of familial HLH. A representative NK cell degranulation assay for one of these patients (P19) is shown in **Figure 2C**. To further investigate whether clinical manifestations and a defective NK cell activity were due to mutations in non-encoding regions of *RAB27A* affecting protein expression, we analyzed the presence of RAB27A by Western blot. Indeed, **Figure 2D** (lines 1 to 3) shows that the RAB27A protein was absent or severely reduced in these patients when compared to a healthy donor.

Patients 15, 17 and 18 were carrying a pathogenic homozygous variant in *RAB27A* (c.335del/p.Asn112ThrfsTer3), while this variant was heterozygous in P16. This variant has been previously reported in patients with diagnosis of GS2 and is expected to result in a absent or truncated protein product (14, 34). As expected, NK cell degranulation in NK cells from P15, P17 and P18 was defective consistent with the genetic study. P16 also displayed a defective NK cell degranulation despite presenting the pathogenic variant in heterozygous condition (see below). P22 presented two heterozygous variants, c.335del (p.Asn112ThrfsTer3) and c.333A>C (p.Arg111Ser). The variant p. Asn112ThrfsTer3 was also found in P15, P17 and P18. In contrast, the variant p.Arg111Ser has not been reported in individuals with GS2. The variant has a very low frequency (gnomAD 0.00003%; Hom=0). A defective NK cell degranulation observed in this patient suggest that this novel variant can be classified as likely pathogenic.

**Figure 3:**
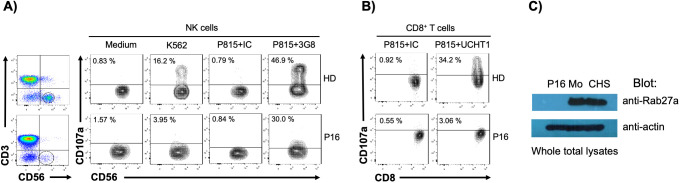
Impaired NK cell and CTL degranulation in a patient with a heterozygous *RAB27A* variant. **(A)** FACS plots illustrating the expression of CD107a on NK cell surface (CD3-CD56+) using PBMCs from a healthy donor (HD) or from P16 after incubation with medium or with NK-sensitive K562 target cells. Alternatively, PBMCs were stimulated with P815 cells in the presence of an isotype control (IC) or anti-CD16 antibody (clone 3G8). **(B)** FACS plots illustrating the expression of CD107a in cytotoxic T lymphocytes (CTLs) using PBMCs from a healthy donor or from P16. PBMCs were activated with phytohemagglutinin plus IL-2 for 48 h and then stimulated with P815 cells in the presence of an isotype control (IC) or anti-CD3 antibody (clone UCHT1). Data are presented as the percentage of CTLs expressing surface CD107a. **(C)** Immunoblot analysis of Rab27a protein expression and beta actin in whole-cell lysates from PBMCs of P16 patient (Pt), the patient’s mother, and a patient with Chediak-Higashi syndrome.

In P16 the variant c.335del was heterozygous. Degranulation in both NK cells and cytotoxic CD8+ T lymphocytes was impaired (**Figures 3A, B**). Since GS is an autosomal recessive disorder, a heterozygous variant in RAB27A by itself could not explain a defective NK cell degranulation in cytotoxic lymphocytes. All reported patients carrying the c.335del were homozygous or heterozygous in association with a second allelic pathogenic variation in *RAB27A* (30, 35, 36). Therefore, a non-coding variant affecting protein expression not detected by WES was suspected to be present on the other allele. To address this issue, expression of RAB27A was evaluated by western blot revealing absence of RAB27A protein expression in the patient cells in contrast to cells from a healthy donor (mother of patient) and a patient with CHS that showed detectable RAB27A expression (**Figure 3C**). All these data suggest that, in this patient, the defect in NK cell degranulation may be explained by biallelic mutation in RAB27A, one found in the coding *RAB27A* sequence and the other likely present within a non-coding region similarly as in P19, P20 and P21.

Four patients (P23, P24, P25 and P26) with a mutation in LYST were identified. P23 presented the variants c.1897A>T (p.Lys633Ter) and c.6676C>T (p.Arg2226Ter), both already reported in patients with Chédiak-Higashi Syndrome (CHS) (37–39). P24 was a homozygous carrier for the variant in c.3574G>T (p.Glu1192Ter). This mutation creates a premature translational stop signal in the *LYST* gene, which is expected to result in an absent or truncated protein product. This variant is not present in gnomAD nor reported in patients with CHS. An impaired degranulation observed in NK cells from this patient suggest that this variant is likely pathogenic (**Supplementary Figure 1A**). In P26, a homozygous variant c.9784 + 1G>T was identified, that affects a donor splice site in intron 42 of the *LYST* gene. It is expected to disrupt RNA splicing, causing a loss of protein function. While this variant is present in population databases with a weak frequency (rsID rs1345024890, gnomAD AF 0.003%), it is classified as likely pathogenic in ClinVar. NK cells from this patient showed impaired degranulation in a functional assay, confirming that the variant c.9784 + 1G>T is likely pathogenic. P27 presented the homozygous variant c.10222G>A (p.Gly3408Arg), which has been already reported in the compound heterozygous state in two siblings with CHS (40), however this missense mutation has been classified as a variant of uncertain significance because the lack of functional and genetic evidence. A reverse ADCC in NK cells from the patient displayed a profound defect in NK cell degranulation as compared with NK cells from a healthy control (**Supplementary Figure 1B**). Therefore, our data suggest that this variant can be classified as likely pathogenic. P25 was a heterozygous carrier for the variant c.10900G>A (rs1291892090, p.Val3634Met) which was private (not found in gnomAD). This patient was diagnosed with systemic idiopathic juvenile arthritis (sIJA) and presented episodes of active HLH. Since no NK degranulation assays were performed for this patient, this variant remains as of uncertain significance.

In P28, two compound heterozygous variants in *AP3B1* were identified, c.1679A>G (p.Asn560Ser) and c.2018A>G (p.Lys673Arg). Both variants are present in population databases (rs776064198, gnomAD 0.08%; and rs763619135 gnomAD 0.08% respectively) but have not been reported in the literature in individuals affected with AP3B1-related conditions. A NK cell degranulation assay displayed a defective NK cell function suggesting that both variants can affect NK cell function (**Supplementary Figure 2**).

**Figure 4:**
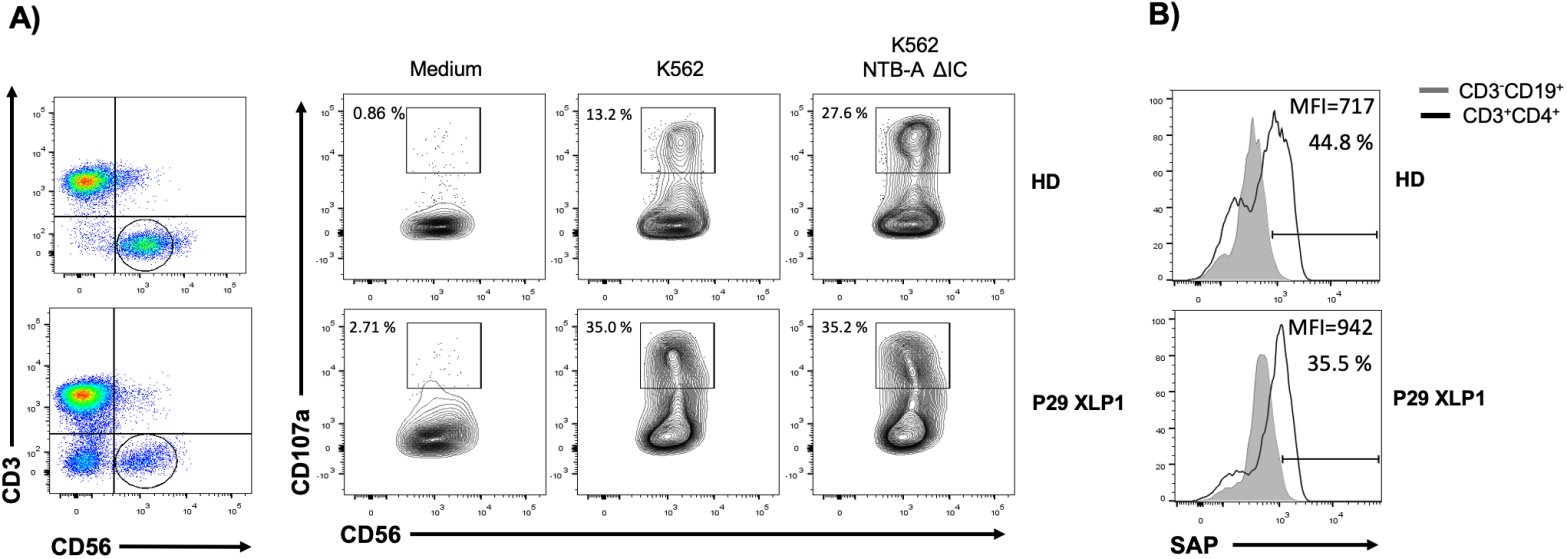
Enhanced NK cell degranulation but normal SAP expression in a patient with XLP1. **(A)** FACS plots illustrating the expression of CD107a on NK cell surface (CD3-CD56+) using PBMCs from a healthy donor (HD) or from P29 after incubation with medium, parental K562 target cells, or K562 expressing NTB-A. **(B)** FACS histograms depicting frequencies of SAP+ CD4+ T lymphocytes from a healthy donor (Upper histogram) or from P29 (lower histogram). Filled histogram represent the SAP staining in CD19^+^ B cells. Open histogram represent the SAP staining in CD4^+^CD3^+^ T cells.

P29 was a hemizygous carrier for a variant in SH2D1A, c.293T>C (p.Leu98Pro). This variant is no present in population databases (gnomeAD no frequency) but has been observed in individuals with X-linked lymphoproliferative syndrome (XLP) (27, 41, 42). However, since no molecular studies have been provided, this missense mutation has been classified as a variant of uncertain significance. Here we have analyzed, by flow cytometry, the ability of NK cells to degranulate and the SAP protein expression. As shown in **Figure 4A**, in response to K562 cells, NK cells from the patient displayed a significant increase in the frequencies of CD107a-expressing NK cells when compared to a healthy control. The significant increase of NK cells to degranulate in the absence of SAP expression towards nonhematopoietic cells or K562 cells, which lack SLAM family receptor expression, has been reported previously (43). Moreover, when K562 cells expressing NTB-A, lacking the cytoplasmic domain, where used as target, we observed that NK cells from healthy donor displayed a significant increase in NK cell degranulation when compared to parental K562. In contrast, such increase was not observed for NK cells form patient. These assays suggest that NK cells expressing the SAP variant p.Leu98Pro displayed an abnormal NK cell function. Interestingly, when we analyzed the SAP protein expression in CD4+ T cells from patient (**Figure 4B**), we did not observe any difference in the frequencies of SAP-expressing T cells when compared to T cells from healthy donor, suggesting that albeit this missense mutation does not affect the protein stability protein, it may cause a deleterious effect on cell signaling.

Patients 30 and 31 displayed normal NK cell degranulation when compared to healthy controls. Although these patients presented at least one HLH episode that required hospitalization, no variants in FLH-related genes were found by further directed-exome sequencing, which may explain why NK degranulation was found to be normal in these patients (Data not shown). However, each patient presented with a distinctive heterozygous variant in TREX1, which encodes the major DNA exonuclease in mammals. Further studies are required to investigate whether variants in TREX1 are sufficient to trigger HLH episodes.

## Discussion

This work highlights the importance of incorporating functional NK cell degranulation assays as a first-line diagnostic tool in patients presenting with clinical criteria for HLH. Our findings demonstrate that CD107a-based degranulation assays provide a rapid, cost-effective screening method that effectively distinguishes primary forms of HLH affecting cytotoxic lymphocyte function from secondary forms, allowing for timely therapeutic decisions while genetic confirmation is pursued.

Our cohort of 31 patients underscores the genetic heterogeneity underlying HLH syndromes in the Mexican population. We identified fifteen previously unreported variants across multiple genes (*UNC13D, STX11, LYST, RAB27A, STXBP2*, and *AP3B1*), expanding the mutational spectrum associated with familial HLH and related syndromes. Novel variants include missense, nonsense and deletion mutations. The mechanisms by which these missense mutations impact on the proper function of their respective proteins need to be determined in further studies. It is important to note that a clear limitation of our study was the lack of definitive evidence, such as rescue experiments, to validate the pathogenicity of all identified novel variants. Consequently, all previously unreported variants in our cohort were classified as likely pathogenic according to the ACMG guidelines. In addition, we observed that beyond a potential association between the detected genetic variants and clinical outcomes, external variables—such as timely medical intervention, the presence of EBV/CMV triggers, and transplant waiting times—were also important drivers of patient prognosis.

Notably, the detection of non-coding mutations affecting RAB27A expression, as has been previously reported (44, 45), emphasizes that negative genetic screening of coding regions does not exclude a monogenic cause when functional assays demonstrate a clear NK cell dysfunction. Moreover, mutations in noncoding RNAs, promoters, enhancers, deep intronic sequences, and untranslated regions (UTRs) can alter splicing, transcript stability, or gene regulation, compromising normal gene activity. Such variants, typically missed by exome sequencing, account for up to 50% of all molecular diagnoses in monogenic illnesses. In addition to resolving molecular diagnoses, whole-genome sequencing (WGS) can also broaden the spectrum of variants that cause familial hemophagocytic lymphohistiocytosis. Furthermore, these findings highlight the importance of integrating WGS into the standard workflow to identify potential variants outside coding areas, as has been recently advised (46). The identification of heterozygous variants in genes typically associated with autosomal recessive HLH, particularly in the context of systemic inflammatory conditions such as sJIA, supports the emerging concept that monoallelic mutations may act as predisposing risk factors for secondary HLH in hyperinflammatory settings (47, 48).

In resource-limited settings where next-generation sequencing may not be immediately accessible, the combination of NK cell degranulation assays with targeted protein expression studies (such as Western blot for RAB27A or perforin) provides a pragmatic diagnostic algorithm that may help to distinguish familial forms of HLH from secondary HLH observed in the context of hyperinflammatory settings such as sJIA or as a result of viral infections other than EBV. This approach not only facilitates early diagnosis and appropriate treatment initiation but also helps identify patients who may benefit from definitive therapies such as hematopoietic stem cell transplantation.

In conclusion, functional immunological assessment remains an indispensable complement to genetic testing in the diagnostic evaluation of HLH. The integration of both approaches enables accurate classification of HLH subtypes, guides therapeutic management, and contributes to our understanding of the complex immunological and genetic landscape underlying hyperinflammatory syndromes.

## Data Availability

The raw data supporting the conclusions of this article will be made available by the authors, without undue reservation, to any qualified researcher upon reasonable request.
