# Supplementary material for: Genetic and molecular approaches for patients with familial hemophagocytic lymphohistiocytosis: a multi-center experience from Mexico
Source: Front Immunol. 2026 Jul 3;17:1849014. doi: 10.3389/fimmu.2026.1849014 (PMC13392799; doi:10.3389/fimmu.2026.1849014)
Supplement: Supplementary file 1 [file DataSheet1.pdf]

Patient	Gene	Variant	Gender	onset	Clinical manifestation	Treatment	Transplanted/ Outcome
P1	*PRF1*<br>*UNC13D*	c.755A>G (p.Asn252Ser) c.445G>A (p.Gly149Ser) c.71G>A (p.Arg24His)	Male	NDA	NDA	NDA	Transplanted/ Alive
P2	*PRF1*	c.445G>A (p.Gly149Ser) Hom	Female	4 mo	Scarlet fever. Positive Scarlet fever. Positive *mycoplasma pneumoniae* serology (IgM). serology (IgM). Persistent fever, hypotonia, seizures, neck rigidity, hepatomegaly, pancytopenia, and Hb 9.8 g/dL. AST 913 U/L, ALT 936 U/L.	Clarithromycin. Paracetamol.	Non-Transplanted/ Deceased
P3	*UNC13D*	c.859-3C>A Hom	Female	8 mo	Persistent fever, hepatosplenomegaly, hypertriglyceridemia (1,280 mg/dL), thrombocytopenia, Hb 8.61 g/dL, hyperferritinaemia (2,026 ng/mL), D-dimer 1,131 ng/mL, and monocytosis (4,300 cells/μL). Erythema and edema at BCG inoculation site. At the age of 1 mo oral candidiasis.	Nystatin and amoxicillin. TMP/SMX and itraconazole. Ceftriaxone, bezafibrate and omeprazole. Antipyretic therapy and systemic steroid.	Non-Transplanted/ Deceased
P4	*UNC13D*	c.1072dup (p.Ser358Lysfs*103) **Novel** c.859del (p.Arg287Glufs*41)	Female	1 yo 15 do	Persistent fever, vomit, diarrhea, abdominal distention, pancytopenia, hyperferritinaemia, hypofibrinogenemia, hepatosplenomegaly, and seizures. Hemophagocytosis in bone marrow.	TMP/SMX and antipyretic therapy. Cyclosporine, dexamethasone, and etoposide. IVIG.	Non-Transplanted/ Alive
P5	*UNC13D*	c.570-2A>G c.2795T>C, (p.Leu932Pro) **Novel**	Male	12 yo 6 mo	Jaundice and fever, cervical lymphadenopathy, hepatosplenomegaly, cytopenia, hypertriglyceridemia, hypercholesterolemia, hypofibrinogenemia, D-dimer 1,306 ng/mL, Hb 7.4 g/dL, and hyperferritinaemia (3,870 ng/mL). ALT 354 U/L, AST 370 U/L, and GGT 1,707 U/L. Metabolic syndrome, systemic hypertension, and cholestatic hepatitis. EBV infection (2,771 copies/mL) HLH triggered by EBV.	TMP/SMX and fluconazole. Cyclosporine, dexamethasone, and etoposide. IVIG. Hydralazine, amlodipine, tramadol, phytomenadione, hydroxyzine, metoclopramide, sertraline, omeprazole, ibuprofen, and racecadotril.	Non-Transplanted/ Deceased

P6	*UNC13D*	c.833T>C (p.Leu278Pro) Hom **Novel**	Female	NDA	NDA	NDA	NDA
P7	*UNC13D*	c.1831G>C (p.Ala611Pro) Hom **Novel**	Male	1 yo 3 mo	HLH sepsis-associated, upper respiratory infection (hospitalization), then, pneumonia (enterovirus and metapneumovirus detection) accompanied with gastroenteritis. Hypertriglyceridemia, hypercholesterolemia, hyperferritinaemia (9,650 ng/mL), thrombocytopenia, hypertriglyceridemia, hypofibrinogenemia, elevated liver transaminase levels, and D-dimer 2,239 ng/mL. EBV infection (1,598 UI/mL), negative for CMV.	TMP/SMX. Cyclosporine, dexamethasone, and etoposide. IVIG.	Non-Transplanted/ Alive
P8	*UNC13D*	c.1721C>G (p.Ser574Ter) Hom **Novel**	Female	3 mo	Short stature, persistent fever, hepatosplenomegaly, anemia, neutropenia, hypertriglyceridemia, hypofibrinogenemia, hyperferritinaemia (2,261 ng/mL), and thrombocytopenia. Systemic hypertension, meningoencephalitis, focal epilepsy, oral candidiasis, and hepato-splenic candidiasis. ALT 199 U/L and AST 126 U/L. Hemophagocytosis in bone marrow. Positive EBV, CMV, and parvovirus serology.	Cefotaxime, paracetamol, pantoprazole, enalapril, and levetiracetam. TMP/SMX and itraconazole. Ruxolitinib. Methylprednisolone. Cyclosporine, dexamethasone, and etoposide. IVIG.	Non-Transplanted/ Deceased
P9	*UNC13D*	c.859del (p.Arg287Glufs*41) c.2795T>C (p.Leu932Pro) **Novel**	Male	NDA	NDA	NDA	Non-Transplanted/ Deceased
P10	*STX11*	c.83C>A, p.S28* Hom **Novel**	Male	NDA	NDA	NDA	Non-Transplanted/ Deceased
P11	*STX11*	c.157_160del (p.Asp53Lysfs*9) Hom **Novel**	Female	3 mo	Persistent fever, pancytopenia, hepatosplenomegaly, hypertriglyceridemia (490 mg/dL), hypofibrinogenemia (48 mg/dL), hyperferritinaemia (10,300 ng/ml), encephalopathy, Hb 6.1	TMP/SMX, antipyretic therapy amlodipine, and hydralazine. Methylprednisolone. Cyclosporine, dexamethasone, and etoposide. IVIG.	Transplanted/ Alive

					g/dL, and hyperbilirubinemia. Dysmegakaryopoiesis. Positive EBV and CMV (10 682 copies/mL) infection.		
P12	*STXBP2*	c.284del (p.Pro95Argfs*24) **Novel** c.560C>T (p.Pro187Leu)	Male	1 mo	Severe lymphopenia, neutropenia, and Hb 8.1 g/dL. Transplanted at two months of age.	TMP/SMX, fluconazole, and filgrastim. IVIG. Cyclosporine, dexamethasone, and etoposide. Intrathecal administration of methotrexate and hydrocortisone.	Transplanted/ Alive
P13	*STXBP2*	c.284del (p.Pro95Argfs*24) Het	Female	2 mo	Persistent fever, Hb 7.4 g/dL, vomit, abdominal distention, irritability, diarrhea, and septic shock. Hypertriglyceridemia (391 ng/mL), hyperferritinaemia (8,200 ng/mL), neutropenia, and D-dimer 5,791 ng/mL. Hepatosplenomegaly, cytopenia, systemic inflammation, seizures, and chronic hepatitis.	Antipyretic therapy. Cyclosporine, dexamethasone, and etoposide. IVIG. Intrathecal administration of methotrexate and hydrocortisone.	Non-Transplanted/ Deceased
P14	*RAB27A*	c.343A>G (p.Ser115Gly)N Hom **Novel**	Female	From birth	Pancytopenia, hepatosplenomegaly, hypertriglyceridemia (301 ng/mL), hyperferritinaemia (947 ng/mL), Hb 8.2 g/dL, and silvery-gray hair. Hemophagocytosis and pneumonia.	Broad-spectrum antibiotics and IVIG. Cyclosporine and dexamethasone. TMP/SMX and fluconazole.	Transplanted/ Deceased
P15	*RAB27A*	c.335del (p.Asn112Thrfs*3) Hom	Female	2 mo	Persistent fever, silvery-gray hair, sialorrhea, vomit, and seizures. Diffuse subarachnoid hemorrhage, granulomatous brain lesions, invasive aspergillosis, and systemic inflammation. Hb 7.2 g/dL, pancytopenia, hypertriglyceridemia, hyperferritinaemia, and hemophagocytosis in bone marrow	Antipyretic therapy. Cyclosporine, dexamethasone, and etoposide. Voriconazole and IVIG.	Non-Transplanted/ Deceased
P16	*RAB27A*	c.335del (p.Asn112Thrfs*3) Het	Male	2 yo 4 mo	Persistent fever, diarrhea, recurrent respiratory infections, bronchiolitis, hepatosplenomegaly, and neutropenia. HLH triggered by EBV.	Antipyretic therapy. Cyclosporine, dexamethasone, and etoposide. Paracetamol, diphenhydramine, ondansetron,	Non-Transplanted/ Alive

						hydrocortisone, and IVIG.	
P17	*RAB27A*	c.335del (p.Asn112Thrfs*3) Hom	Female	NDA	NDA	NDA	NDA
P18	*RAB27A*	c.335del (p.Asn112Thrfs*3) Hom	Female	NDA	NDA	NDA	NDA
P19	*RAB27A*	Not found in encoding region	Female	4 yo 4 mo	Persistent fever, asthenia, adynamia, and weight loss.<br>Bilateral foot ecchymosis, hepatomegaly, thrombocytopenia, anemia, and jaundice.<br>Hemophagocytosis in bone marrow.<br>Lesions on the hands and feet with desquamation.<br>Positive EBV serology.	Ciprofloxacin, clindamycin, and ibuprofen.<br>Dexamethasone and etoposide.<br>Dicloxacillin.	Non-transplanted/ Alive
P20	*RAB27A*	Not found in encoding region	Female	1 yo 3 mo	Persistent fever, pancytopenia, Hb 9.3 g/dL, hepatosplenomegaly, elevated liver transaminase levels, neurological deterioration, upper respiratory infections, molluscum contagiosum, maculopapular lesions, sudden blindness, seizures, and silvery-gray hair.<br>Negative for EBV and CMV infection.	Ceftriaxone, vancomycin, teicoplanin, and fluconazole.<br>Diphenylhydantoin and then, levetiracetam.<br>Methylprednisolone, cyclosporine, prednisone, dexamethasone, itraconazole, mycophenolic acid, phenytoin, ranitidine, and TMP/SMX.<br>IVIG.	Transplanted (Failed)/ Alive
P21	*RAB27A*	Not found in encoding region	Female	10 mo	Abdominal distention, persistent fever, pancytopenia, hepatosplenomegaly, hypofibrinogenemia (90 mg/dL), hypertriglyceridemia (505 ng/mL), hyperferritinaemia (2982 ng/mL), hyperbilirubinemia, dermatosis, diarrhea, silvery-gray hair, scaly papules, and hyperpigmented macules,<br>Hemophagocytosis in bone marrow.<br>Hb 5,9 g/dL, ALT 70 U/L, AST 61 U/L, and GGT 151 U/L.	Antipyretic therapy.<br>Ceftriaxone, dexamethasone, cyclosporine, and etoposide.<br>Furosemide and enalapril.<br>TMP/SMX and fluconazole.<br>IVIG.	Transplanted (Failed)/ Deceased
P22	*RAB27A*	c.335del (p.Asn112Thrfs*3)	Male	10 yo 4 mo	Persistent fever, cytopenia, hepatosplenomegaly,	Antipyretic therapy.	Non-transplanted/ Alive

		c.333A>C (p.Arg111Ser) **Novel**			hyperferritinaemia, hypofibrinogenemia, and elevated liver transaminase levels. EBV infection (162 copies/mL). Hemophagocytosis in bone marrow.	Cyclosporine, dexamethasone, and etoposide. TMP/SMX, amlodipine, cholecalciferol, and calcium carbonate.	
P23	*LYST*	c.1897A>T (p.Lys633*)/ c.6676C>T (p.Arg2226*)	Male	2 mo	Neutropenia, thrombocytopenia, anemia, hypercholesterolemia (331 mg/dL), hyperferritinaemia (1,500 ng/mL), silvery-gray hair, and pneumonia leading to sepsis. Recurrent upper respiratory infections. Pharyngitis and fever.	NDA	Non-transplanted/ Deceased
P24	*LYST*	c.3574G>T (p.Glu1192*) Hom **Novel**	Male	6 mo	Persistent fever, neutropenia, thrombocytopenia, and hemophagocytosis in bone marrow. EBV infection (435,130 copies/mL). Cervical lymphadenopathy.	Paracetamol, ciprofloxacin, and vancomycin. Dexamethasone and etoposide.	Non-transplanted/ Deceased
P25	*LYST*	c.10900G>A (p.Val3634Met) Het	Female	From birth	Persistent fever, lymphopenia hepatosplenomegaly, Hb 8.5 g/dL, hyperferritinaemia (2,303 ng/mL), AST 52 U/L, hyperfibrinogenemia (455 mg/dL), and hypertriglyceridemia (233 mg/dL). Positive CMV serology. Atopic dermatitis, chronic urticaria, arthralgia, myalgia, and acute vasculitis. Soluble CD25 11,370 pg/mL.	Paracetamol and ibuprofen. Ceftriaxone, methylprednisolone and then prednisolone. Cyclosporine, dexamethasone, and etoposide. Ruxolitinib. Methotrexate and tocilizumab.	Non-transplanted/ Alive
P26	*LYST*	c.9784+1G>T Hom **Novel**	Male	NDA	NDA	NDA	NDA
P27	*LYST*	c.10222G>A (p.Gly3408Arg) Hom **Novel**	Male	8 yo	Generalized hyperpigmentation (since 3 yo). Pancytopenia, anemia, hepatosplenomegaly, hepatitis, cervical adenopathy, hypoalbuminemia, persistent fever, hyperferritinaemia (3,000 ng/mL), and hypertriglyceridemia (219 ng/mL).	Dexamethasone, prednisone, and cyclosporine. TMP/SMX.	Non-transplanted/ Alive

P28	*AP3B1*	c.1679A>G (p.Asn560Ser)/ **Novel** c.2018A>G (p.Lys673Arg) **Novel**	Male	From birth	Jaundice, total bilirubin 30 mg/dL, Hb 5.05.g/dL, cholestatic syndrome, microcytic anemia, hepatosplenomegaly, leukocytosis, thrombocytopenia, isolated macroplatelets, hyperferritinaemia (1,784 ng/mL), hypoalbuminemia, and hydrocele. AST 1,278 U/L and ALT 327 U/L.	NDA	Non-transplanted/ Deceased
P29	*SH2D1A*	c.293T>C (p.Leu98Pro)	Male	8 yo	Postnatal failure to thrive. Recurrent respiratory infections, severe pneumonia, chronic cough, and muscle weakness. Extrapulmonary tuberculosis. Hepatosplenomegaly, thrombocytosis, hypogammaglobulinemia, cervical and supraclavicular adenopathy, abdominal distention, and septic shock. Autopsy findings: disseminated non-Hodgkin lymphoma.	Rifampicin, isoniazid, pyrazinamide, and ethambutol. Furosemide, paracetamol, cefepime, and TMP/SMX. IVIG. Meropenem, vancomycin, and omeprazole.	Non-transplanted/ Deceased

EBV: Epstein-Barr Virus
CMV: Cytomegalovirus
HLH: hemophagocytic Lymphohistiocytosis
BCG: Bacille Calmette-Guérin
AST: Aspartate aminotransferase
ALT: Alanine aminotransferase
GGT: Gamma-glutamyl transferase
TMP/SMX: Trimethoprim/sulfamethoxazole
IVIG: Intravenous Immunoglobulin
Hb: Hemoglobin
yo: years-old
mo: months-old
do: days-old
NDA: No data available

Supplementary Table 1

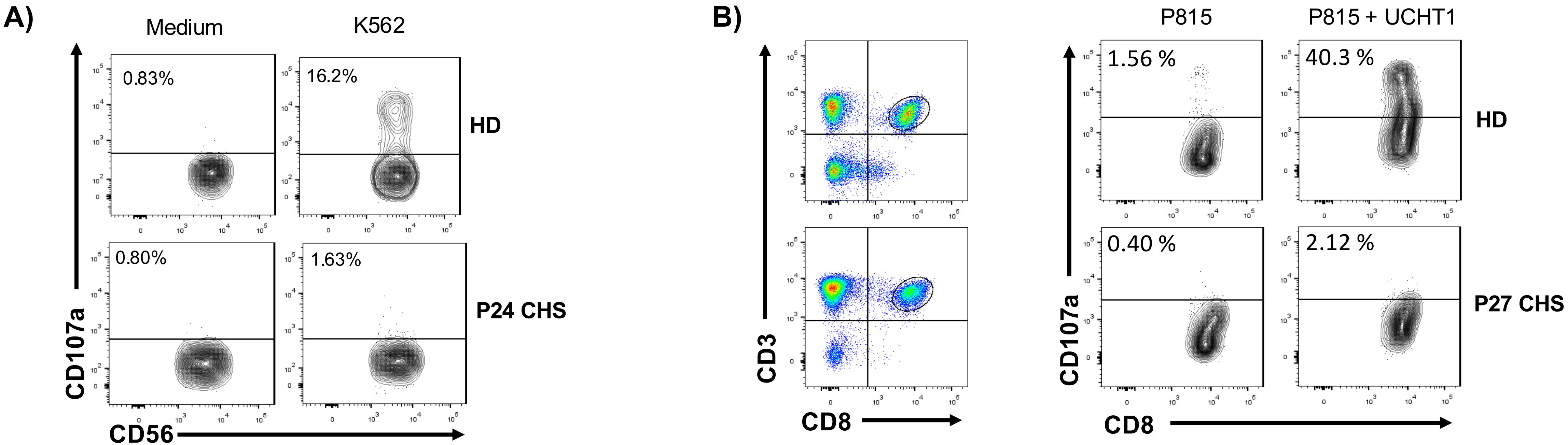

Supplementary Figure 1

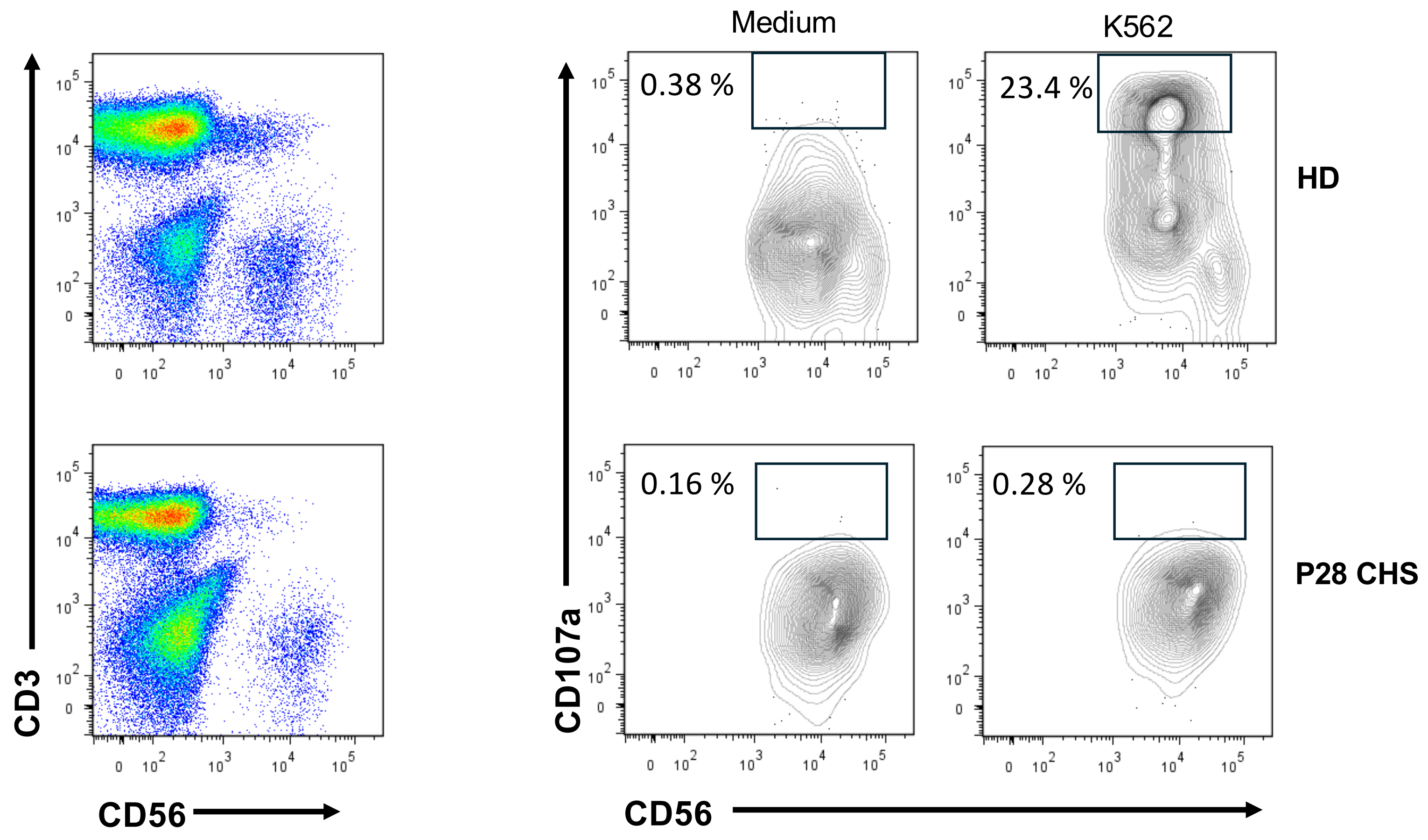

Supplementary Figure 2

Supplementary Figure 1. (A) FACS plots illustrating the expresion of CD107a on NK cell surface (CD3-CD56+) using PBMCs from a healthy donor (HD) or a patient with CHS (P24) after incubation with medium or with NK-sensitive K562 target cells. Data are presented as the percentage of NK cells expressing surface CD107a. (B) FACS plots illustrating the expresion of CD107a in cytotoxic T lymphocytes (CTLs) using PBMCs from a healthy donor or from P27. PBMCs were activated with phytohemagglutinin plus IL-2 for 48 h and then stimulated with P815 cells in the presence of an isotype control (IC) or anti-CD3 antibody (clone UCHT1). Data are presented as the percentage of CTLs expressing surface CD107a.

Supplementary Figure 2. (A) FACS plots illustrating the expresion of CD107a on NK cell surface (CD3-CD56+) using PBMCs from a healthy donor (HD) or a patient with Hermansky-Pudlak Syndrome (P28) after incubation with medium or with NK-sensitive K562 target cells. Data are presented as the percentage of NK cells expressing surface CD107a.
